# Supplementary material for: Effect of Physical Activity on Cardiovascular Event Risk in a Population-Based Cohort of Patients with Type 2 Diabetes
Source: Int J Environ Res Public Health. 2021 Nov 24;18(23):12370. doi: 10.3390/ijerph182312370 (PMC8657417; doi:10.3390/ijerph182312370)
Supplement: Supplementary file 1 [file ijerph-18-12370-s001.zip › ijerph-1424756-supplementary.pdf]

International Journal of
Environmental Research and Public Health

MDPI

SUPPLEMENTARY MATERIAL:

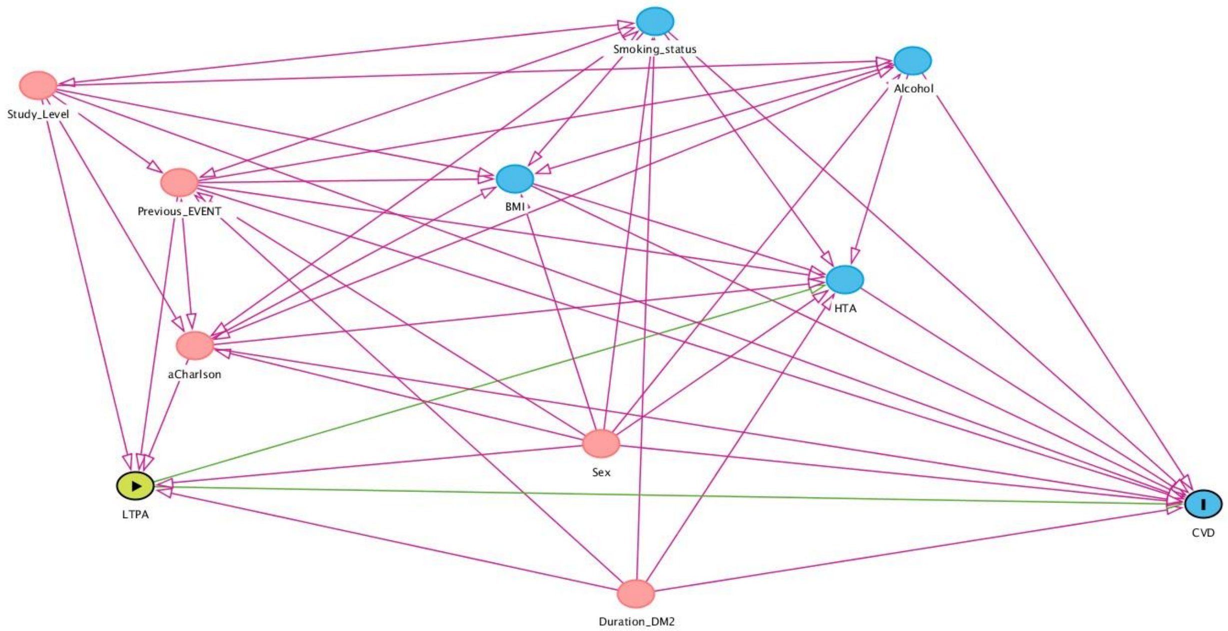

Model adjustment was based on this adapted DAG from Andersen et al. [27]. The minimal sufficient adjustment sets for estimating the effect of PA on CVD was obtained using the web application "DAGitty [28].

Figure S1. Adapted Directed Acyclic Graph (DAG) for model adjustment.

Table S1. Detailed multivariate Cox regression models (Model 3) for both, CVD and all-cause mortality outcomes in the total cohort.

		CVD		All-cause mortality	
**Variable**	**Levels**	**HR (95% CI, *p* Value)**	**Global p**	**HR (95% CI, *p* Value)**	**Global *p***
Gender	Male	Ref.	<0.001	Ref.	<0.001
	Female	0.75 (0.66–0.85, $p < 0.001$)		0.66 (0.60–0.72, $p < 0.001$)	
Education Level	No	Ref.	0.745	Ref.	0.064
	Primary School	0.96 (0.86–1.07, $p = 0.442$)		0.94 (0.88–1.02, $p = 0.123$)	
	High School	0.97 (0.78–1.21, $p = 0.805$)		0.84 (0.69–1.01, $p = 0.063$)	
	University	0.93 (0.69–1.25, $p = 0.618$)		0.84 (0.67–1.06, $p = 0.146$)	
Duration of T2D		1.02 (1.01–1.02, $p < 0.001$)	<0.001	1.01 (1.00–1.01, $p = 0.002$)	<0.001
History of CVD	No	Ref.	<0.001	Ref.	<0.001
	Yes	1.60 (1.43–1.79, $p < 0.001$)		1.16 (1.07–1.26, $p < 0.001$)	
Smoking_status	Non-smoker	Ref.	<0.001	Ref.	<0.001
	Ex-smoker	0.96 (0.84–1.10, $p = 0.556$)		1.07 (0.97–1.18, $p = 0.185$)	
	Smoker	1.31 (1.12–1.53, $p = 0.001$)		1.24 (1.10–1.41, $p = 0.001$)	
Alcohol group	No	Ref.	0.236	Ref.	0.002
	Yes	0.93 (0.83–1.05, $p = 0.237$)		0.89 (0.82–0.98, $p = 0.012$)	
BMI		0.99 (0.98–1.00, $p = 0.006$)	0.219	0.98 (0.98–0.99, $p < 0.001$)	0.112
HTA	No	Ref.	0.013	Ref.	0.522
	Yes	1.13 (0.99–1.29, $p = 0.070$)		0.95 (0.86–1.04, $p = 0.242$)	
aCharlson	< 55	2.48 (1.94–3.16, $p < 0.001$)	<0.001	2.41 (1.83-3.17, $p < 0.001$)	<0.001
	55-65	1.74 (1.55–1.95, $p < 0.001$)		1.90 (1.69–2.13, $p < 0.001$)	
	65-75	1.54 (1.45–1.65, $p < 0.001$)		1.52 (1.44–1.61, $p < 0.001$)	

	75-85	1.34 (1.28–1.41, $p < 0.001$)		1.33 (1.28–1.38, $p < 0.001$)	
	>85	1.22 (1.13–1.31, $p < 0.001$)		1.21 (1.16–1.26, $p < 0.001$)	
Physical Activity	Inactive	Ref.	<0.001	Ref.	<0.001
	Partially.active	0.87 (0.74–1.03, $p = 0.097$)		0.75 (0.67–0.83, $p < 0.001$)	
	Active	0.72 (0.61–0.84, $p < 0.001$)		0.51 (0.46–0.57, $p < 0.001$)	

HR: Hazard ratios.

Table S2. Characteristics of cases (fatal and non-fatal CVD) and controls included in nested case-control study by CVD history.

		No CVD history			With CVD history		
**Variable**	**Levels**	**Controls**	**Cases**	***p* Value** $^{\delta}$	**Controls**	**Cases**	***p* Value** $^{\delta}$
Total *N* (%)		2195 (66.1)	1128 (33.9)		1837 (65.3)	976 (34.7)	
Age	Mean (SD)	73.5 (10.8)	73.6 (10.9)	0.932	76.7 (8.6)	76.7 (8.8)	0.916
Gender	Male	1260 (57.4)	650 (57.6)	0.932	1142 (62.2)	605 (62.0)	0.958
	Female	935 (42.6)	478 (42.4)		695 (37.8)	371 (38.0)	
Education Level	No	847 (38.6)	436 (38.7)	0.899	785 (42.7)	411 (42.1)	0.404
	Primary School	1209 (55.1)	617 (54.7)		962 (52.4)	506 (51.8)	
	High School	116 (5.3)	62 (5.5)		60 (3.3)	39 (4.0)	
	University	23 (1.0)	13 (1.2)		30 (1.6)	20 (2.0)	
Duration T2D	Median (IQR)	8.0 (5.0–12.0)	8.0 (5.0–12.0)	0.497	9.0 (6.0–12.0)	9.0 (6.0–13.0)	0.532
Smoking_status	Non-smoker	1332 (63.1)	636 (59.4)	<0.001	1061 (60.2)	561 (60.5)	0.866
	Ex-smoker	467 (22.1)	209 (19.5)		531 (30.1)	272 (29.3)	
	Smoker	313 (14.8)	225 (21.0)		170 (9.6)	94 (10.1)	
Alcohol group	No	1349 (65.6)	708 (66.4)	0.695	1193 (68.8)	653 (70.8)	0.301
	Yes	708 (34.4)	359 (33.6)		541 (31.2)	269 (29.2)	
BMI	Mean (SD)	30.1 (5.1)	30.0 (5.5)	0.642	30.4 (6.0)	29.8 (4.7)	0.009
HTA	No	553 (25.2)	256 (22.7)	0.122	344 (18.7)	157 (16.1)	0.091
	Yes	1642 (74.8)	872 (77.3)		1493 (81.3)	819 (83.9)	
aCharlson	1-2	1166 (53.1)	588 (52.1)	0.790	403 (21.9)	215 (22.0)	0.708
	3-4	882 (40.2)	452 (40.1)		974 (53.0)	505 (51.7)	
	5-6	147 (6.7)	88 (7.8)		460 (25.0)	256 (26.2)	
Physical Activity	Inactive	230 (10.5)	155 (13.7)	0.001	291 (15.8)	185 (19.0)	0.001
	Partially	695 (31.7)	376 (33.3)		646 (35.2)	373 (38.2)	
	Active	1270 (57.9)	597 (52.9)		900 (49.0)	418 (42.8)	

$^{\delta}$ X^2 test used for comparison except for Study level, aCharlson and Physical Activity, for which Test for trend in proportions was used

Table S3. Characteristics of cases (all-cause mortality) and controls included in nested case-control study by CVD history.

		No CVD history			With CVD history		
**Variable**	**Levels**	**Controls**	**Cases**	***p* Value** $^{\delta}$	**Controls**	**Cases**	***p* Value** $^{\delta}$
Total *N* (%)		5063 (65.9)	2621 (34.1)		3664 (65.1)	1962 (34.9)	
Age	Mean (SD)	78.4 (9.7)	78.5 (9.7)	0.794	80.7 (7.7)	80.9 (7.9)	0.500
Gender	Male	2657 (52.5)	1382 (52.7)	0.855	2028 (55.3)	1087 (55.4)	0.992
	Female	2406 (47.5)	1239 (47.3)		1636 (44.7)	875 (44.6)	
Education Level	No	2282 (45.1)	1178 (44.9)	0.360	1851 (50.5)	983 (50.1)	0.990
	Primary School	2547 (50.3)	1306 (49.8)		1721 (47.0)	912 (46.5)	
	High School	143 (2.8)	84 (3.2)		67 (1.8)	47 (2.4)	
	University	91 (1.8)	53 (2.0)		25 (0.7)	20 (1.0)	

Duration of T2D	Median (IQR)	9.0 (5.0 to 12.0)	9.0 (5.0 to 12.0)	0.766	10.0 (6.0 to 14.0)	10.0 (6.0 to 14.0)	0.584
Smoking status	Non-smoker	3360 (69.0)	1640 (65.7)	<0.001	2394 (67.7)	1244 (66.4)	0.110
	Ex-smoker	998 (20.5)	496 (19.9)		899 (25.4)	472 (25.2)	
	Smoker	514 (10.6)	362 (14.5)		241 (6.8)	157 (8.4)	
Alcohol group	No	3310 (69.3)	1756 (71.6)	0.042	2543 (73.2)	1418 (76.4)	0.011
	Yes	1468 (30.7)	696 (28.4)		931 (26.8)	437 (23.6)	
BMI	Mean (SD)	29.6 (5.5)	29.3 (6.1)	0.070	29.9 (5.8)	29.6 (5.9)	0.132
HTA	No	1095 (21.6)	686 (26.2)	<0.001	670 (18.3)	389 (19.8)	0.170
	Yes	3968 (78.4)	1935 (73.8)		2994 (81.7)	1573 (80.2)	
aCharlson group	1-2	2266 (44.8)	1154 (44.0)	0.261	582 (15.9)	319 (16.3)	0.701
	3-4	2526 (49.9)	1303 (49.7)		2090 (57.0)	1090 (55.6)	
	5-6	271 (5.4)	164 (6.3)		992 (27.1)	553 (28.2)	
Physical Activity	Inactive	635 (12.5)	537 (20.5)	<0.001	687 (18.8)	591 (30.1)	<0.001
	Partially.active	1731 (34.2)	1005 (38.3)		1412 (38.5)	774 (39.4)	
	Active	2697 (53.3)	1079 (41.2)		1565 (42.7)	597 (30.4)	

$^{\delta}$ X^2 test used for comparison except for Study level, aCharlson and Physical Activity, for which Test for trend in proportions was used

Table S4. Unadjusted and adjusted ORs (95% CI) of CVD (fatal and non-fatal CVD) and mortality for physical activity by CVD history.

	**CVD**						**All-Cause Mortality**					
	**No CVD History**			**With CVD History**			**No CVD History**			**With CVD History**		
**PA**	**OR**	**95% CI**	***p***	**OR**	**95%CI**	***p***	**OR**	**95% CI**	***p***	**OR**	**95% CI**	***p***
**Model1** [1]												
Inactive	Ref			Ref			Ref			Ref		
Partially active	0.79	[0.62;1.01]	0.064	0.94	[0.75;1.17]	0.578	0.67	[0.58;0.77]	<0.001	0.63	[0.55;0.73]	<0.001
Active	0.68	[0.54;0.86]	0.001	0.74	[0.59;0.92]	0.006	0.44	[0.38;0.50]	<0.001	0.42	[0.36;0.49]	<0.001
**Model2** [2]												
Inactive	Ref			Ref			Ref			Ref		
Partially active	0.87	[0.61;1.24]	0.430	0.80	[0.58;1.12]	0.192	0.78	[0.63;0.98]	0.032	0.65	[0.51;0.82]	0.001
Active	0.76	[0.53;1.08]	0.127	0.62	[0.45;0.87]	0.005	0.52	[0.41;0.65]	< 0.001	0.46	[0.36;0.58]	<0.001

[1] Model1 covariates: Physical Activity (PA); [2] Model2 covariates: smoking status, alcohol intake, body mass index, hypertension and PA.
